# Acceptance of and satisfaction with online educational classes through the technology acceptance model (TAM): the COVID-19 situation in Korea

**DOI:** 10.1007/s12564-021-09716-7

**Published:** 2021-09-15

**Authors:** Jee-Hoon Han, Hye Ji Sa

**Affiliations:** Department of Sport Industry Studies, Yonsei University, Seoul, Republic of Korea

**Keywords:** Technology acceptance model, Online learning, Distance education, COVID-19

## Abstract

This study examines the current state of acceptance of online classes using the technology acceptance model. The background of the study is the turning point in Korean education in response to the COVID-19 pandemic and speculation about changes in the post-COVID educational environment. To measure the acceptance rate of online classes, a survey was conducted on a total of 313 university students taking online classes. The data were analyzed using structural equation modeling. The results of the study are as follows: First, the perceived ease of use of online classes showed a positive effect on perceived usefulness. Second, both the perceived ease of use and usefulness of online classes showed a positive effect on educational satisfaction. Third, both the perceived usefulness and satisfaction showed a positive effect on the acceptance intention of online education. However, the perceived ease of use did not have a positive effect on acceptance intention. These results suggest that satisfaction with online education can be further improved by developing online classes that are easy to use, focusing on the features that are frequently used by university students. In addition, universities should continuously provide training and advice to increase students’ perceived usefulness of online classes.

## Introduction

The international community is facing challenging times because of the coronavirus disease 2019 (COVID-19). The worsening economic and social disorder is not only being witnessed in the medical field but also across all areas of society. Consequently, in March 2020, the World Health Organization (2020) declared COVID-19 a pandemic, placing it at the highest risk level for infectious diseases.

COVID-19 has forced numerous organizations to modify their systems and strategies and adopt new technologies. Most organizations did not have adequate time to reflect on the methods of introducing and integrating these new strategies and related technologies into their existing settings and processes (Carroll & Conboy, 2020). Universities around the world were no exception to this. Like many other aspects of daily life, COVID-19 is severely impacting students, instructors, and educational institutions worldwide (Mailizar et al., 2020). Its global spread has led to the total suspension of public education and university classes, forcing schools to revise their original education plans (Toquero, 2020). This is not the first time that traditional educational activities have been suspended in response to an infectious disease. Previously, viruses such as SARS (2004) and H1N1 (2009) adversely impacted existing educational activities in numerous countries (Cauchemez et al., 2014). Many countries have resolved to promote online education using softwares such as Zoom and Webex to counter the disruption that COVID-19 has had on education and restore the normal educational order.

Today, online education is an important teaching method; it has developed worldwide quickly and is becoming a key mode of instruction for educators. Educational institutions are actively exploring how to effectively teach students through the Internet and provide them with valuable experiences. Even before COVID-19, education technology had already experienced remarkable growth and extensive development; global education tech investment reached $18.46 billion in 2019, and the total online education market is forecasted to reach $350 billion by 2025. Unsurprisingly, given this increase, the use of language-related apps, virtual tutors, video conferencing tools, and online learning software has soared since the COVID-19 outbreak (World Economic Forum, 2020).

COVID-19 has given online classes and non-face-to-face distance education a new form and meaning. First, owing to the unpredictable nature and rapid spread of the pandemic, online education systems were developed within a short period of time without sufficient review at the national level. Under a broad social consensus, most people implicitly agreed to the online education systems. Despite rapid development around the globe, online education has only served as a basic infrastructure used to supplement regular school education (Mishra et al., 2020). There were few previous scenarios in which a large-scale online curriculum had been applied in response to situations such as a pandemic and, therefore, no universities were fully prepared transition to online education. As such, short-term decisions and processes were implemented across all areas of education and for all students (Ock, 2020).

In accordance with the Korean government’s strong social distancing measures, after postponing the opening of schools four times in March 2020, the South Korean Ministry of Education finally decided to allow online learning, leading to the full implementation of online classes. Prior to this, universities, which have many foreign students as compared to elementary, middle, and high schools had already initiated online learning and fully implemented online classes. The non-face-to-face online classes conducted by schools largely consist of real-time interactive classes that mainly use lecture- and activity-type contents and are based on performance of assignments (Ministry of Education, 2020). Depending on subject characteristics and the instructor’s circumstances, each university can autonomously choose one of these methods or combine two or more to conduct classes. Consequently, teaching methods have become more complex than ever, meaning that class quality and satisfaction can greatly differ with the instructor or subject. In particular, online classes in the first semester began abruptly due to the unprecedented spread of COVID-19. As a result, instructors did not have adequate preparation time (e.g., procuring ICT for their online classes) and students also faced difficulties adapting to the new classroom environment (Lee & Kim, 2020). Asarbakhsh and Sandars (2013) also reported that system failure during teaching, disconnection of video and voice systems, and difficulties with using the technology affected educational satisfaction.

Changes in the educational environment due to COVID-19 have become clear, and the world of education reached a turning point in response to these changes in the post-COVID era. Ha (2020) recommends that schools’ online platforms (learning management systems) be rapidly improved, and Toquero (2020) asserts that it is important for academic institutions to improve their curricula and use new teaching methods and strategies, noting the importance of using technology in education. Furthermore, although students were unable to receive help from friends in classrooms and labs or even access facilities such as libraries and gymnasiums (Patricia, 2020), online education has helped prevent the spread of COVID-19 and allowed school curricula to continue being taught (Mishra et al., 2020).

Universities now need innovative strategies to ensure the continuity of education for students (Zhu & Liu, 2020). Arguably, simply moving in-person lectures online is not innovative. Developing and utilizing personalized learning systems alongside online learning are the core elements of educational innovation. Personalized online learning systems redefine professor–student interactions, and genuine innovation can only be achieved if their educational outcomes exceeded those of in-person lectures. In terms of implementing online classes and utilizing the learning data they produce, new approaches to education are needed to cultivate talent that can positively influence the Fourth Industrial Revolution (4IR). At the same time, learning institutions utilizing 4IR technology can be used to ascertain whether universities are adapting to the changes being brought about by the 4IR.

In this context, this study aims to investigate the current state of acceptance of online classes using the technology acceptance model (TAM) and to explore changes in education trends after COVID-19.

## Theoretical background

### Technology acceptance model

Davis (1989) expanded Fishbein and Ajzen’s (1977) theory of reasoned action (TRA) to propose the TAM. Currently, the TAM is recognized as the best model for understanding the acceptance of information technology (Gefen & Straub, 2000; Venkatesh & Davis, 2000; Wang et al., 2003).

Davis’s (1989) TAM is presented as a concise and useful theoretical framework for investigating how perceived usefulness and perceived ease of use of a new technology or service affect its acceptance. As a result, while incorporating the causality of TRA, TAM uses the two concepts of “perceived ease of use” and “perceived usefulness” to explain users’ intention to use information systems (Davis, 1989).

Davis (1989) defines perceived ease of use, a variable that affects the acceptance and adoption of a particular information technology (computer technologies, services, software, etc.), as the extent to which a user feels that the system is easy to understand and use (Rose & Fogarty, 2006). It can also be described as an individual’s subjective perception that using a particular system is effortless (Rigopoulos & Askounis, 2007). Therefore, perceived usefulness can be defined as the degree to which a person believes that using a particular system would enhance his or her job performance (Davis, 1989).

In TAM, perceived ease of use is a preceding variable of perceived usefulness (Davis, 1989). This is because the easier a user feels it is to use a new technology or service, the more useful they perceive it to be. Additionally, if one technology is perceived as more convenient than another, consumers are likely to use it more often. Hence, TAM provides a useful analytic framework for the use and adoption of information technology through the variables of perceived usefulness, perceived ease of use, and use intention.

Davis’s (1989) research spread rapidly and was applied to the acceptance of different types of information technology. When TAM was first introduced, it was a theory explaining the acceptance and definition of new information technology. Since then, it has been put to use in various fields, including new technologies and services (Venkatesh, 2006). As the most widely used model for information technology acceptance research (King & He, 2006), it has been frequently applied in studies on e-commerce and Internet acceptance (Gefen & Straub, 2000) and has made it possible to explain user acceptance of other researchers’ information technologies (Venkatesh et al., 2003). After Davis’s (1989) TAM was validated, research into external variables that influence perceived usefulness and perceived ease of use began (Shih, 2004).

### Educational satisfaction

Educational satisfaction is the overall evaluation of and emotional response to educational services (Choi, 2005), and can be described as the difference between a student’s expectations and actual perceptions of the educational services provided by a school (Park et al., 2002). Student satisfaction after receiving education is regarded as an important variable for measuring educational outcomes and results to help revise or improve educational content (Athiyaman, 1997).

Oldfield and Baron (2000) opine that since educational satisfaction is based on judgments of the educational services provided by educational institutions and instructors, the quality of lectures and interactions with professors has a significant influence on students’ educational satisfaction. Astin (1980) describes educational satisfaction as students’ subjective response to an educational experience and further opines that students’ satisfaction can indicate directions for enhancing the quality and effectiveness of education. Similarly, online educational satisfaction—the learner’s subjective view of his or her online education experience—implies that online education’s quality and results meet or exceed the learner’s expectations (Spreng & Mackoy, 1996). Class satisfaction in online education is complex and multifaceted; it not only serves as a driving force enabling learners to continue learning without interruption, but it also plays an important role in improving learning outcomes by promoting learners’ participation (Palmer & Holt, 2009). Therefore, online educational satisfaction can be regarded as an essential factor for enhancing educational outcomes because it is a variable representing the educational outcomes themselves.

In this study, university students are recognized as customers who consume educational services and customer satisfaction—seen as a combination of the customer’s cognitive judgment and emotional response (Oliver, 1977)—was used to define the degree of satisfaction and acceptance of educational services provided to university students, who are the customers. Research measuring educational satisfaction has revealed the market and customer orientations needed to gain an economic advantage in the university market by recognizing students as consumers and satisfying them (Lee, 1998). In this context, customer satisfaction refers to a conscious and cognitive evaluation and judgment of the purchased service. Therefore, it can be defined as both a psychological state and an outcome of connecting the customer’s emotions before and after a purchase (Oliver, 1981). While the concepts and definitions of customer satisfaction vary among scholars, the most common approach for explaining customer satisfaction is that satisfaction increases if performance exceeds the customer’s expectations in their post-purchase evaluation of performance, whereas satisfaction decreases if performance is lower than expected (Oliver, 1980). Hunt (1977) describes customer satisfaction as an explicit evaluation that the consumer’s experience exceeded their minimum expectations, while Oliver (1993) defines customer satisfaction as a process formed by multidimensional human emotions. Jeong (2012) also defines customer satisfaction as a multidimensional concept that includes customer emotions that persist even after all service experience processes and evaluations have been completed.

Since both the subjective experience of the service user and the service provider’s objective quality play a role in determining customer satisfaction, educational outcomes should equally be evaluated on the premise of customer satisfaction. Furthermore, regardless of how customer satisfaction is measured, it should ultimately be determined by the customers’ perceived level of the educational services based on their expectations.

### Online education

Distance education or online learning can be defined as a method that enhances rapid learning by applying information and Internet technology to distribute educational content. The “e” in e-learning stands for electronic, efficient, exploratory, experiential, expanded, easy to use, and enhanced (Zhou et al., 2020).

The global spread of COVID-19 has led to the suspension of in-person classes for over 850 million students. This has resulted in online education gaining more attention as schools were forced to transition to online learning. Consequently, researchers are conducting various studies on online learning, which can be largely divided into research on students’ learning perceptions and research on the changed educational environment.

First, regarding research on online learning and students’ learning perceptions, studies in various fields related to online education have focused on enhancing students’ online learning experience (Al-Samarraie et al., 2018; Sun et al., 2008), and studies on learners’ perceptions of online learning are also underway (Wei & Chou, 2020). Research on whether students’ perceptions of online learning are related to learning outcomes is also in progress (Ke & Kwak, 2013). For example, Bertea (2009) and Morris (2011) report that university students’ perceptions and experiences of the Internet can have a decisive influence on online learning outcomes. On the other hand, Sahin and Shelley (2008) suggest that online class satisfaction would improve if students were able to use online tools and perceived distance education to be a useful and flexible way of learning, communicating, and sharing information.

These studies demonstrate that online learning provides learners with a more flexible and convenient learning environment that enables self-directed and customized learning. Moreover, through synchronous and asynchronous communication technologies, online learning can improve not only interactions between students, instructors, and peers, but also the quality and quantity of learning. Put simply, the positive characteristics that learners perceive in online learning environments consist of class flexibility, interactions with friends and instructors, time and location flexibility, and easy access to a variety of online content and expertise.

Numerous studies have also examined how educational environments have changed during this pandemic period. Bao (2020) explains how universities have had to transition from classroom-based education to online education as the pandemic spreads. Mishra et al. (2020) also conducted an empirical study in India to identify students’ views on online education during the COVID-19 pandemic, and various studies are under way to investigate specific situations in various countries (e.g., Bojović et al., 2020; Patricia, 2020).

Among the environmental factors, the use of technology in particular requires examination. To evaluate readiness for online learning, other aspects such as the technical efficiency of using digital devices, self-control abilities, and Internet browsing skills must be considered. Dray et al. (2011) developed a scale for students to self-assess their readiness for online learning. The scale measures four dimensions: basic technology skills to use specific applications (e.g., email, Internet, spreadsheets, and documents); access to technology, including ownership of devices and connectivity to the Internet; usage of technology, such as the nature and frequency of use; and relationship with ICT, such as beliefs, values, confidence, and comfort with technology. Keramati et al. (2011) also conducted a study investigating the role of readiness factors in the relationship between online learning factors and outcomes, and found that readiness factors moderate this relationship. This implies that online learning readiness can include multifaceted concepts such as the technical efficiency of computer usage and technology usage (Hung, 2016; Hung et al., 2010; Keramati et al., 2011; McVay, 2000).

Therefore, based on previous studies, the present study used TAM to investigate the acceptance intention of and satisfaction with online classes and ascertain students’ technology acceptance and readiness for online learning.

### Research questions and hypotheses

**Fig. 1:**
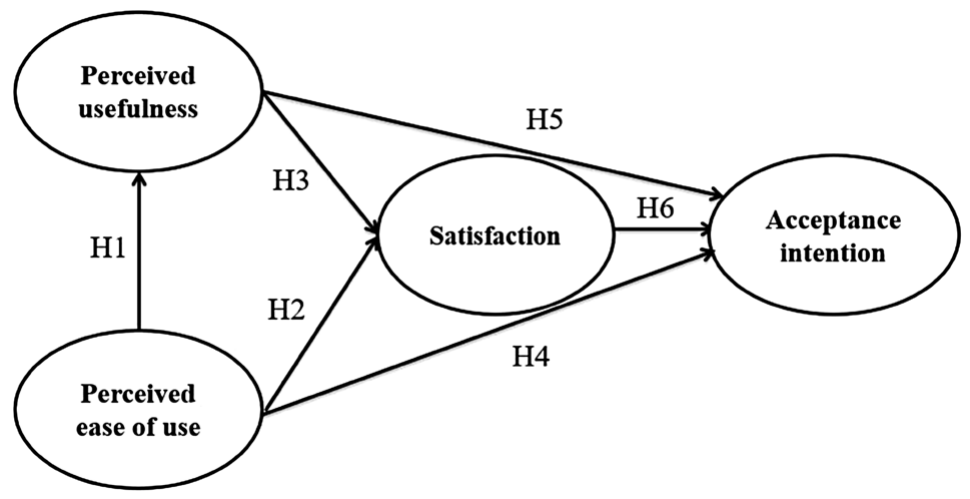
Research model

Based on a review of previous TAM-related studies, various hypotheses were developed for the variables of perceived ease of use, perceived usefulness, satisfaction, and acceptance intention, as described below. The independent variables are perceived ease of use and perceived usefulness, and the dependent variables are satisfaction and acceptance intention. The hypotheses are shown in Fig. 1 below.

#### Relationship between TAM and satisfaction

Davis (1989) suggested that perceived ease of use and perceived usefulness were directly affected as factors for the use of information technology. A study on the Social Network Services (SNS) acceptance of a club by Korean professional baseball fans suggested that perceived ease of use has a positive effect on perceived usefulness (Bae et al., 2015). In addition, Joo (2018) notes that both the perceived ease of use and perceived usefulness of a specialized tourism platform have a positive effect on customer satisfaction. Based on the preceding studies, this study develops the following hypotheses:

##### Hypothesis 1:

Perceived ease of use of online classes has a positive effect on perceived usefulness.

##### Hypothesis 2:

Perceived ease of use of online classes has a positive effect on educational satisfaction.

##### Hypothesis 3:

Perceived usefulness of online classes has a positive effect on educational satisfaction.

#### Relationship between TAM and acceptance intention

Lu et al. (2010) study found that perceived usefulness and perceived ease of use are the main variables that increase the intention to use. In addition, a study by Jackson et al. (1997) suggests that perceived usefulness and perceived ease of use are important factors for continued intention to use. In a preliminary study based on the Korean context, Chun et al. (2017) found that perceived ease of use and perceived usefulness positively influence the intention to accept a matching service for sports participants. Byun et al. (2015) also found that perceived ease of use and usefulness of sports-related apps had a positive effect on acceptance intention. Based on these studies, hypothesis 4 is as follows:

##### Hypothesis 4:

Perceived ease of use of online classes has a positive effect on acceptance intention.

#### Relationship between satisfaction and acceptance intention

Gil et al. (2011) suggest that educational satisfaction has a positive effect on positive behavioral intentions, and Jang and Kim (2017) also suggest that it has a positive effect on intention to use in SNS. In Lee et al.’s (2014) study, user satisfaction of the horse riding experience was found to be a factor that positively influences behavioral intention. These studies confirm a positive relationship between satisfaction and intention to use. Thus, hypotheses 5 and 6 are as follows:

##### Hypothesis 5:

Perceived usefulness of online classes has a positive effect on acceptance intention.

##### Hypothesis 6:

Educational satisfaction with online classes has a positive effect on acceptance intention.

## Research methods

### Research subjects

To investigate university students’ satisfaction with and acceptance intention of online classes, the required sample size for the structural equation model in this study was calculated using the “A-priori Sample Size Calculator for Structural Equation Models” available online (Soper, 2021). Convenience sampling—a sampling method where members of a target population who are easily accessed by researchers are included in a study (Etikan et al., 2016)—was used. In this study, the population was all university students in Korea currently taking online classes due to the COVID-19 pandemic.

Data were collected through an online questionnaire survey of university students taking distance/online classes, who were selected through convenience sampling, from December 9 to December 16, 2020. The respondents were recruited through EM-Brain, a company specializing in online surveys. The survey was emailed to registered users of the company. The contents of the questionnaire and the purpose of the research were provided to each recipient prior to the completion of the questionnaire. Only recipients who confirmed that they were enrolled as college students and were currently taking online classes were allowed to complete the questionnaire. The survey was terminated for those who responded they that were not college students or did not take online classes. All the responses were anonymous. The target sample for this study was 671 students; however, only 313 fully completed the survey.

Details on the 313 students are shown in Table 1 below. They comprised 156 men and 157 women; 50 first years (16%), 70s years (22.4%), 96 third years (30.7%), and 97 fourth years (31%). In terms of their educational programs, 104 were humanities and social science majors (33.2%), 137 natural science majors (43.8%), 21 education majors (6.7%), 30 medicine and health majors (9.6%), and 21 other majors (6.7%). Home (dormitory) was the most common location for taking classes (285, 91.1%). A total of 70.9% respondents indicated that they had been taking online classes for a period of one to two semesters, while 48.2% took online classes three to four times per week, which were the most common responses.

**Table 1:** Characteristics of participants

Characteristics		*n*	Percentage (%)
Gender	Male	156	49.8
	Female	157	50.2
Year	First year	50	16.0
	Second year	70	22.4
	Third year	96	30.7
	Fourth year	97	31.0
Major	Humanities and social sciences	104	33.2
	Natural sciences and engineering	137	43.8
	Education	21	6.7
	Health and medicine	30	9.6
	Other	21	6.7
Class method	Only online classes	163	52.1
	Both online and offline classes	150	47.9
Location for taking online classes	Home (dormitory)	285	91.1
	Cafe	19	6.1
	Library	7	2.2
	Other	2	.6
Period	< 1 Semester	9	2.9
	1 Semester	41	13.1
	1–2 Semesters	222	70.9
	> 2 Semesters	41	13.1
Frequency of lessons per week	1–2 times	33	10.5
	3–4 times	151	48.2
	5 or more times	129	41.2
Total		313	100

### Measurement tools

Perceived usefulness, perceived ease of use, and acceptance intention were used with reference to the measurement items of previous studies that are applicable to the current conditions in Korea (Byun et al., 2015; Chun et al., 2017; Joo, 2018) and the characteristics of the humanities and social sciences, based on TAM, as developed and extended by Agarwal and Karahanna (2000) and Venkatesh and Davis (2000). For customer satisfaction, the factors used in Oliver (1977) were revised and supplemented to align them to Korean sentiments (Gil et al., 2011). Finally, this study used four perceived usefulness items, four perceived ease of use items, six customer satisfaction items, and four acceptance intention items.

### Factor analysis and reliability verification

The content validity, construct validity, and model fit of the measurement tools were verified separately. First, the content validity of the survey was verified by one researcher and one professor teaching an online class, after which confirmatory factor analysis was performed to confirm construct validity and model fit.

For the relative fit indices, the model was assessed using the comparative fit index (CFI) and the non-normed fit index, also known as the Tucker–Lewis index (TLI). The absolute fit index was assessed using the root mean square error of approximation (RMSEA). TLI and CFI above 0.90 and RMSEA between 0.08 and 0.10 indicate a good model fit (Hu & Bentler, 1999; MacCallum et al., 1996). TLI was 0.954, CFI was 0.963, and RMSEA was 0.076, which satisfied the model fit criteria. Reliability was verified through Cronbach’s α coefficient, and the value was greater than 0.70, confirming the reliability of the measurement tool (Nunnally & Bernstein, 1994; Van de Ven & Ferry, 1980). Additionally, construct reliability (CR) and average variance extracted (AVE) were calculated for all factors to verify convergent validity. CR and AVE were at least 0.60 and 0.50, respectively, thus confirming validity (Bagozzi &Yi, 1988). Details of the reliability analysis are provided in Table 2 below.

**Table 2:** Result of Confirmatory factor and reliability analyses

Factor	Question	Standardized estimate	Error variation	CR	AVE	Cronbach’s α
Perceived usefulness	1. If I use online classes, I will be able to efficiently obtain educational information	.842	.243	.908	.713	.896
	2. If I use online classes, I will be able to obtain useful and interesting educational information	.839	.247			
	3. The educational information obtained through online classes will be very useful	.865	.197			
	4. I will be able to improve my educational outcomes through online classes	.787	.432			
Perceived ease of use	1. I will be able to clearly understand how to use this online class	.675	.584	.887	.665	.876
	2. I will be able to skillfully use this online class	.824	.289			
	3. Learning how to use online classes will be easy	.880	.194			
	4. Online classes will be easy to use	.844	.251			
Satisfaction	1. I am satisfied with choosing this online class	.928	.158	.884	.666	.949
	2. I am satisfied with using this online class	.948	.121			
	3. I am satisfied with this online class	.895	.232			
	4. I approve of the fee for this online class	.548	.922			
Acceptance intention	1. I intend to accept online classes in the future	.867	.280	.933	.779	.892
	2. I will use online classes in the future	.866	.277			
	3. I will talk positively about online classes to others in the future	.929	.181			
	4. I will recommend using online classes to others in the future	.929	.175			

*χ*^2^ = 270.123, df = 97, CFI = .963, TLI = .954, RMSEA = .076

*CR* construct reliability, *AVE* average variance extracted, *χ*^2^ chi-square, *df* degrees of freedom, *CFI* comparative fit index, *TLI* Tucker–Lewis index, *RMSEA* root mean square error of approximation

### Data processing

Data analysis was performed using SPSS and AMOS software. First, a frequency analysis was performed to examine the demographic characteristics of the survey participants. Confirmatory factor analysis was then conducted for each item to verify the validity and reliability of the survey tool, and Cronbach’s α coefficient was calculated to ensure internal consistency between the items. Moreover, a correlation analysis was performed to investigate the correlation and multicollinearity of each factor with the confirmed unidimensionality of satisfaction and acceptance intention with TAM. To test the research hypotheses, a path analysis using structural equation modeling was performed, through which each hypothesis was either supported or rejected.

## Results

### Correlation analysis between factors

A correlation analysis was conducted to ascertain the relationship between university students’ satisfaction with online classes and the acceptance intention of the classes. In TAM, perceived usefulness and perceived ease of use consisted of two sub-factors, while satisfaction and acceptance intention consisted of one factor. Table 3 shows the correlation analysis results. Pearson’s product-moment correlation coefficient did not indicate any issues with multicollinearity, as the correlation coefficients between latent variables did not exceed 0.80.

**Table 3:** Correlation analysis among factors

	1	2	3	4
Perceived usefulness	1			
Perceived ease of use	.537**	1		
Satisfaction	.682**	.442**	1	
Acceptance intention	.713**	.520**	.848**	1

1 = Perceived usefulness, 2 = Perceived ease of use, 3 = Satisfaction, 4 = Acceptance intention

**p < .01

### Research model fit

The structural model of the university students’ satisfaction with and acceptance intention of online classes was analyzed, and the results are shown in Table 4 using maximum likelihood (ML) as a parameter. The CFI was 0.962 (> 0.90), TLI was 0.953 (> 0.90), and RMSEA was 0.077 (< 0.10), which satisfied the model fit criteria.

**Table 4:** Model fit using maximum likelihood as a parameter

*χ*^2^	df	CFI	TLI	RMSEA
272.057	96	.962	.953	.077

*χ*^2^ chi-square, df degrees of freedom, *CFI* comparative fit index, *TLI* Tucker–Lewis index, *RMSEA* root mean square error of approximation

### Hypothesis verification

**Fig. 2:**
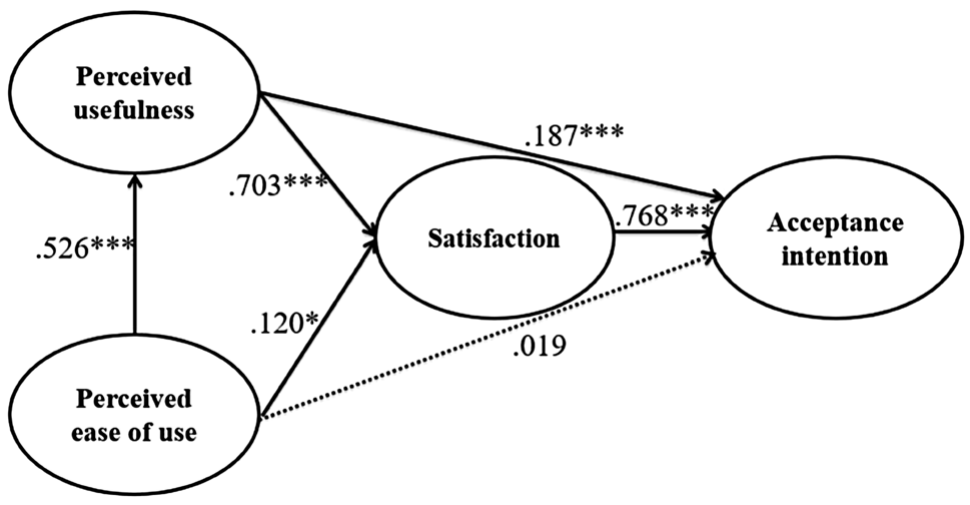
Path analysis result

Table 5 and Fig. 2 show the verification results of the research hypotheses set in the research model to identify the relationship between university students’ satisfaction with online classes and the acceptance intention of the classes through TAM.

**Table 5:** Path analysis results

H	Path	Path coefficient	SE	*t*	Supported/rejected
H1	Perceived ease of use → Perceived usefulness	.526	.089	7.378***	Supported
H2	Perceived ease of use → Satisfaction	.120	.285	2.239*	Supported
H3	Perceived usefulness → Satisfaction	.703	.076	11.701***	Supported
H4	Perceived ease of use → Acceptance intention	.019	.053	.553	Rejected
H5	Perceived usefulness → Acceptance intention	.187	.065	3.549***	Supported
H6	Satisfaction → Acceptance intention	.768	.053	14.115***	Supported

*SE* standard error

*p < .05, ***p < .001

The analysis of Hypothesis 1 (“The perceived ease of use of online classes has a positive effect on perceived usefulness”) resulted in a path coefficient of 0.526 and a *t*-value of 7.378, which indicate statistical significance. Therefore, Hypothesis 1 was supported (*p* < 0.001). The analysis of Hypothesis 2 (“The perceived ease of use of online classes has a positive effect on satisfaction”) resulted in a path coefficient of 0.120 and a *t*-value of 2.239, which indicate statistical significance. Therefore, Hypothesis 2 was supported (*p* < 0.05). The analysis of Hypothesis 3 (“The perceived usefulness of online classes has a positive effect on educational satisfaction”) resulted in a path coefficient of 0.703 and a *t*-value of 11.701, which indicate statistical significance. Therefore, Hypothesis 3 was supported (*p* < 0.001). The analysis of Hypothesis 4 (“The perceived ease of use of online classes has a positive effect on acceptance intention”) resulted in a path coefficient of 0.019 and a *t*-value of 0.553, which indicate that it was not statistically significant. Therefore, Hypothesis 4 was rejected. The analysis of Hypothesis 5 (“The perceived usefulness of online classes has a positive effect on acceptance intention”) resulted in a path coefficient of 0.187 and a *t*-value of 3.549, which indicate statistical significance. Therefore, Hypothesis 5 was supported (*p* < 0.001). The analysis of Hypothesis 6 (“Educational satisfaction with online classes will have a positive effect on acceptance intention”) resulted in a path coefficient of 0.768 and a *t*-value of 14.115, which indicate statistical significance. Therefore, Hypothesis 6 was supported (*p* < 0.001).

## Discussion

The objective of this study was to analyze university students’ educational satisfaction with and acceptance of online classes using TAM. According to the results, perceived ease of use showed a statistically positive effect on perceived usefulness and educational satisfaction, but it had no statistically positive effect on acceptance intention. Furthermore, perceived usefulness had a statistically positive effect on educational satisfaction and acceptance intention, and educational satisfaction had a statistically positive effect on acceptance intention. These findings are further discussed below.

First, perceived ease of use had a positive effect on perceived usefulness. This represents the basic relationship proposed by TAM. A meta-analysis of 88 previous studies using TAM presented by King and He (2006) reported that perceived ease of use has a positive influence on perceived usefulness, and this was also confirmed by the present study. In another meta-analysis, Schepers and Wetzels (2007) found that 51 out of 53 studies (96.23%) on the relationship between perceived ease of use and perceived usefulness reported a significant influence. Anuar and Othman (2012) also reported that since perceived ease of use and perceived usefulness have a positive relationship, it can be inferred that users perceive easy-to-use systems as more useful. Moreover, Gumussoy and Calisir (2009) reported that if a new technology is easy to use and does not take long to learn, real users’ efficiency increases; therefore, online educational services should be designed to be easy to use. These results suggest that the outcomes of online education can be enhanced by making online educational services easy to use.

Second, the results show that perceived ease of use and perceived usefulness had a positive effect on educational satisfaction. The higher the perceived ease of use and perceived usefulness, the higher the educational satisfaction. Numerous previous studies have suggested that perceived ease of use and usefulness have a positive effect on user satisfaction. For instance, in a study of users of computer-based accounting systems, Seddon (1997) performed an empirical analysis on the effect of system usefulness on user satisfaction and reported a significant relationship. Researchers have also found that perceived usefulness has a positive effect on user satisfaction in the information systems field (Bhattacherjee, 2001; Hong et al., 2006; Lin et al., 2005; Oghuma et al., 2016). Landrum and Prybutok (2004) suggested that perceived usefulness has a very strong influence on satisfaction, and in a study on information systems, Bhattacherjee (2001) demonstrated that online banking users’ perceived usefulness positively influences their satisfaction. In a study on the technical characteristics of forming and building a health information website, Kim and Chang (2007) observed that as more reliable and diverse information was provided, the website’s efficiency and usefulness increased and its users’ customer satisfaction improved. Roca et al. (2006) applied an extended TAM to investigate the continuous use intention of e-learning information technology, and their findings revealed that perceived usefulness and ease of use are positively related to user satisfaction. Furthermore, as system linkage and information searching become easier, customer satisfaction with websites increases (Liao et al., 2007). In a study on smartphone purchases using an extended TAM, Agrebi and Jallais (2015) found that perceived ease of use and perceived usefulness positively influenced user satisfaction.

Based on the aforementioned empirical results, this paper suggests that these findings can form a theory applicable to the expanded online education industry that provides educational content to university students. For instance, when university students encounter a new platform providing educational services, the easier it is to use, the higher the students’ satisfaction will be. In other words, when using online educational platforms, university students’ satisfaction increases if they can easily find their desired results with just a few clicks. These findings reveal the need to focus on university students’ ease of use by focusing on students’ most frequently used features of online educational services. Additionally, the more university students perceive online educational services as convenient to use, the more their satisfaction is likely to increase. Perceived usefulness means that the time and effort required to use online educational services are reduced, thus making the service more convenient. This study confirmed that university students find it convenient if their desired search results are in one place, accept content that is helpful to them, and feel high levels of satisfaction when this saves time and effort.

Third, the results revealed that perceived ease of use did not have a positive effect on acceptance intention. Although perceived usefulness had a significant effect on the acceptance intention, perceived ease of use did not affect acceptance intention, which is consistent with the findings of Mun et al. (2006) study. On the other hand, perceived usefulness, rather than perceived ease of use, had a significant influence on use intention, which is consistent with Mun et al. (2006) findings that perceived ease of use does not affect acceptance intention. Mun et al. (2006) investigated the adoption of telemedicine technology by Hong Kong physicians and similarly found that perceived ease of use did not affect acceptance intention. However, Mun et al. (2006) suggested that because physicians have a high level of cognitive ability and learning ability, they may still try to adopt new technology despite perceived complexity. Similarly, in this study, students also perceived technology as being complex, but given the need to adopt technology to continue university classes due to COVID-19, the perceived ease of use did not affect students’ acceptance intentions. Hence, it can be inferred that university students do not use online educational services simply because they are easy to use; instead, they use them when they perceive them to be useful. Therefore, in addition to providing online educational services that are convenient to use, universities must ensure that university students perceive the services as useful to promote continuous acceptance intention.

Fourth, the results showed that perceived usefulness had a positive effect on acceptance intention. According to Schepers and Wetzels’ (2007) meta-analysis of TAM, perceived usefulness significantly influenced use intention in 38 prior studies, which is consistent with the present study’s findings. Regarding acceptance intention, according to the theory proposed by Rogers (2003) in “Diffusion of Innovations,” new technologies initially spread to innovators or early adopters and are then accepted in the market as they diffuse in various ways. This acceptance intention stems from an individual’s perception that the technology is useful rather than the objective merits of the information or system environment itself (Daim et al., 2013). Hence, high perceived usefulness leads to high acceptance intention. This corroborates the current study’s findings, which show that a high perception of usefulness of online educational services means that individuals believe they can increase their outcomes or value through it, hence increasing their acceptance intention.

Fifth, the results show that educational satisfaction had a positive effect on acceptance intention. Research has shown that satisfaction after accepting information technology has a positive effect on continuous use intention (Bhattacherjee, 2001). Researchers in various fields are actively investigating the fact that user satisfaction has a positive effect on continuous acceptance intention, such as the use of web portals (Lin et al., 2005), mobile Internet (Hong et al., 2006), web-based services (Lee & Kwon, 2011), and Facebook (Hsu et al., 2014). Reichheld and Schefter (2000) suggest that the positive user experience in an Internet space increases their intention to revisit it. Agrebi and Jallais’ (2015) study also found that user satisfaction has a positive effect on continuous use intention. Accordingly, this study’s finding that high educational satisfaction produces high acceptance intention suggests that improving educational satisfaction results in an in increase in acceptance intention.

To summarize, regarding university students’ long-term educational satisfaction with and acceptance intention of online classes, it is necessary to provide technology and information that can enhance ease of use and usefulness, which can positively influence educational satisfaction and acceptance intention. Moreover, universities should continuously provide education and training to help students perceive online classes as useful.

## Conclusions

This study used TAM to analyze university students’ educational satisfaction with and acceptance of online classes. The results highlighted major factors that impact students’ satisfaction with online classes and technology acceptance. According to the theoretical framework utilized in this study, the education content provided in online classes greatly influences students’ intention to accept online classes. From a theoretical perspective, college students must be satisfied with the education provided and the essential information conveyed through online classes for them to accept online educational services. Therefore, it is important to increase students’ satisfaction with their education given their reluctance to accept online classes. In addition, as a long-term goal, the quality of education in Korea should be improved by implementing new educational methods, instead of replicating existing contents in an online format.

First, a theory was presented on the satisfaction with and acceptance intention of online education as society transitions from the “contactless” era to the “online contact” era. This can serve to facilitate participation in new educational services in these changing times, in which a variety of content and services can be identified. Based on this, universities can satisfy their students by providing useful, easy-to-use educational services, thereby enhancing their competitiveness. Moreover, diverse educational services should be developed to ensure university students’ continuous educational satisfaction and acceptance. These issues should be addressed by universities themselves rather than individual professors. While university professors can freely modify their teaching methods, educational quality should be improved and maintained at the university level. Amidst uncertainty about when and what changes will occur due to COVID-19, the educational quality and satisfaction provided by universities should remain unchanged.

Second, to prepare for the post-COVID era, universities must instill new kinds of knowledge in their students and produce students with non-uniform and diverse capabilities. Therefore, new knowledge and differing perspectives (e.g., computer information processing skills and financial knowledge) must be taught in addition to traditional subjects and competencies such as literacy, numeracy, and critical thinking. For this purpose, it is necessary to provide differentiated, individually customized education and cultivate students’ ability to define and solve problems beyond the knowledge taught in class. Extensible online learning is essential for universities to provide students with broader topics and deeper learning using their current faculty. Any shortcomings of online learning (e.g., limited interaction) should be addressed through core 4IR technologies. Moreover, core 4IR technologies (e.g., virtual reality, augmented reality, and digital twins) that will be further developed are expected to substantially increase the level of online interaction in the near future. Most importantly, adaptive learning (i.e., smart systems—characterized by measurement, data accumulation, analysis, and autonomous responses—applied to education) will open a new horizon of professor–learner interactions that can enhance satisfaction with online classes.

Finally, this study has the following limitations, for which follow-up studies are suggested. First, this study investigated online educational services through classes; however, online classes vary across various fields (humanities, sciences, arts, and physical education) in the ever-changing online education market; they differ in both class characteristics and educational content. Second, this study investigated university education and university students, who use digital devices more frequently and learn how to use them more quickly than other groups. To gain a more in-depth understanding of the kind of educational services required in the “online contact” era, it is necessary to include all groups involved in basic education, which would provide broader findings.

## References

[CR1] Agarwal R, Karahanna E (2000). Time flies when you're having fun: Cognitive absorption and beliefs about information technology usage. MIS Quarterly.

[CR2] Agrebi S, Jallais J (2015). Explain the intention to use smartphones for mobile shopping. Journal of Retailing and Consumer Services.

[CR3] Al-Samarraie H, Teng BK, Alzahrani AI, Alalwan N (2018). E-learning continuance satisfaction in higher education: A unified perspective from instructors and students. Studies in Higher Education.

[CR4] Anuar S, Othman R (2012). Determinants of online tax payment system in Malaysia. International Journal of Public Information Systems.

[CR5] Asarbakhsh M, Sandars J (2013). E-learning: The essential usability perspective. The Clinical Teacher.

[CR6] Astin A (1980). When does a college deserve to be called high quality. Current Issues in Higher Education.

[CR7] Athiyaman A (1997). Linking student satisfaction and service quality perceptions: The case of university education. European Journal of Marketing.

[CR92] Bae, J. S., Won, D. Y., & Cho, K. M. (2015). Factors influencing professional baseball fans acceptance of the social networking service (SNS) provided by the Korean professional baseball teams: From the expanded technology acceptance model perspective. *The Korean Journal of Physical Education, 54*(2), 237–251.

[CR8] Bagozzi RP, Yi Y (1988). On the evaluation of structural equation models. Journal of the Academy of Marketing Science.

[CR9] Bao W (2020). COVID-19 and online teaching in higher education: A case study of Peking University. Human Behavior and Emerging Technologies.

[CR10] Bertea, P. (2009). Measuring students’ attitude towards e-learning: A case study. In *Conference proceedings of eLearning and Software for Education (eLSE)* (No. 01, pp. 417–424). ” Carol I”. National Defence University Publishing House.

[CR11] Bhattacherjee A (2001). Understanding information systems continuance: An expectation-confirmation model. MIS Quarterly.

[CR12] Bojović Ž, Bojović PD, Vujošević D, Šuh J (2020). Education in times of crisis: Rapid transition to distance learning. Computer Applications in Engineering Education.

[CR13] Byun H, Bae JS, Won DY (2015). Factors influencing customers acceptance of the sport branded apps provided by the sport brand company: A case study on Nike running application. Korean Journal of Sport Management.

[CR14] Carroll N, Conboy K (2020). Normalising the “new normal”: Changing tech-driven work practices under pandemic time pressure. International Journal of Information Management.

[CR15] Cauchemez S, Fraser C, Van Kerkhove MD, Donnelly CA, Riley S, Rambaut A, Enouf V, Werf S, Ferguson NM (2014). Middle East respiratory syndrome coronavirus: Quantification of the extent of the epidemic, surveillance biases, and transmissibility. The Lancet Infectious Diseases.

[CR16] Choi KH (2005). An effects of evaluation on the satisfaction and behavioral intention in tourism education. Korean Consumption Culture Association.

[CR17] Chun SB, Lee MC, Lee CW (2017). Analyzing the relationship between leisure constraints negotiation and behavioral intention of O2O sports platform through TAM. The Korean Journal of Physical Education.

[CR18] Daim TU, Basoglu AN, Gunay D, Yildiz C, Gomez F (2013). Exploring technology acceptance for online food services. International Journal of Business Information Systems.

[CR19] Davis FD (1989). Perceived usefulness, perceived ease of use, and user acceptance of information technology. MIS Quarterly.

[CR21] Dray BJ, Lowenthal PR, Miszkiewicz MJ, Ruiz-Primo MA, Marczynski K (2011). Developing an instrument to assess student readiness for online learning: A validation study. Distance Education.

[CR23] Etikan I, Musa SA, Alkassim RS (2016). Comparison of convenience sampling and purposive sampling. American Journal of Theoretical and Applied Statistics.

[CR24] Fishbein M, Ajzen I (1977). Belief, attitude, intention, and behavior: An introduction to theory and research.

[CR25] Gefen D, Straub DW (2000). The relative importance of perceived ease of use in IS adoption: A study of e-commerce adoption. Journal of the Association for Information Systems.

[CR26] Gil HN, Shim SM, Chang HS (2011). The effect of service quality of advanced mercantile management prog. Korean Business Education Review.

[CR27] Gumussoy CA, Calisir F (2009). Understanding factors affecting e-reverse auction use: An integrative approach. Computers in Human Behavior.

[CR28] Ha MJ (2020). Learner reaction to non-face-to-face online lessons. The Korean Society of Cultural and Convergence.

[CR29] Hong S, Thong JY, Tam KY (2006). Understanding continued information technology usage behavior: A comparison of three models in the context of mobile internet. Decision Support Systems.

[CR30] Hsu JS, Lin TC, Tsai J (2014). Does confirmation always matter? Extending confirmation-based theories. Behaviour & Information Technology.

[CR31] Hu LT, Bentler PM (1999). Cutoff criteria for fit indexes in covariance structure analysis: Conventional criteria versus new alternatives. Structural Equation Modeling: A Multidisciplinary Journal.

[CR32] Hung ML (2016). Teacher readiness for online learning: Scale development and teacher perceptions. Computers & Education.

[CR33] Hung ML, Chou C, Chen CH, Own ZY (2010). Learner readiness for online learning: Scale development and student perceptions. Computers & Education.

[CR34] Hunt HK (1977). Conceptualization and measurement of consumer satisfaction and dissatisfaction (No. 77–103).

[CR35] Jackson CM, Chow S, Leitch RA (1997). Toward an understanding of the behavioral intention to use an information system. Decision Sciences.

[CR100] Jang, S. H., & Kim, S. H. (2017). Effects of expectation confirmation, social interaction and perceived usefulness on continuous intention to use SNS in MICE Industry. *The Journal of Information Systems, 26*(3), 211–228.

[CR36] Jeong EY (2012). The impact of encounter service quality in relation to nonverbal communication of flight attendant on brand attitude and customer satisfaction. Journal of Tourism Sciences.

[CR37] Joo SO (2018). The influence of the tour platform on customer satisfaction and behavioral intention by technology acceptance model. International Journal of Tourism Management and Sciences.

[CR38] Ke F, Kwak D (2013). Online learning across ethnicity and age: A study on learning interaction participation, perception, and learning satisfaction. Computers & Education.

[CR39] Keramati A, Afshari-Mofrad M, Kamrani A (2011). The role of readiness factors in E-learning outcomes: An empirical study. Computers & Education.

[CR40] Kim D, Chang H (2007). Key functional characteristics in designing and operating health information websites for user satisfaction: An application of the extended technology acceptance model. International Journal of Medical Informatics.

[CR41] King WR, He J (2006). A meta-analysis of the technology acceptance model. Information & Management.

[CR42] Landrum H, Prybutok VR (2004). A service quality and success model for the information service industry. European Journal of Operational Research.

[CR43] Lee D, Kim M (2020). University students’ perceptions on the practices of online learning in the COVID-19 situation and future directions. Multimedia-Assisted Language Learning.

[CR44] Lee YK (1998). A causal analysis of the influencing factors and consequences of market orientation in junior colleges. Korean Management Review.

[CR45] Lee Y, Kwon O (2011). Intimacy, familiarity and continuance intention: An extended expectation–confirmation model in web-based services. Electronic Commerce Research and Applications.

[CR101] Lee, C. K., Ko, S. K., & Kim, J. O. (2014). Examining structural relationships among horseback riding motivation, value, satisfaction, and behavioral intention. *Korean J Tourism Res, 28*(6), 203–226.

[CR46] Liao C, Chen JL, Yen DC (2007). Theory of planning behavior (TPB) and customer satisfaction in the continued use of e-service: An integrated model. Computers in Human Behavior.

[CR47] Lin CS, Wu S, Tsai RJ (2005). Integrating perceived playfulness into expectation-confirmation model for web portal context. Information & Management.

[CR48] Lu IY, Kuo T, Lee WP (2010). Examining the effects of information quality on behavioral intention of knowledge management system. Journal of Quality.

[CR49] MacCallum RC, Browne MW, Sugawara HM (1996). Power analysis and determination of sample size for covariance structure modeling. Psychological Methods.

[CR50] Mailizar M, Almanthari A, Maulina S, Bruce S (2020). Secondary school mathematics teachers’ views on e-learning implementation barriers during the Covid-19 pandemic: The case of Indonesia. Eurasia Journal of Mathematics, Science and Technology Education.

[CR51] McVay M (2000). How to be a successful distance learning student: Learning on the Internet.

[CR52] Ministry of Education. (2020). Focus on building a distance learning environment for all students. https://www.moe.go.kr/boardCnts/fileDown.do?m=020402&s=moe&fileSeq=16888ec0d9d6f4cf56a71404af510cb7

[CR53] Mishra L, Gupta T, Shree A (2020). Online teaching-learning in higher education during lockdown period of COVID-19 pandemic. International Journal of Educational Research Open.

[CR54] Morris RD (2011). Web 3.0: Implications for online learning. TechTrends.

[CR55] Mun YY, Jackson JD, Park JS, Probst JC (2006). Understanding information technology acceptance by individual professionals: Toward an integrative view. Information & Management.

[CR56] Nunnally JC, Bernstein IH (1994). Psychometric theory.

[CR57] Ock, H. J. (2020, April 22). S. Korea struggles with unprecedented online learning. The Korea Herald. http://news.koreaherald.com/view.php?ud=20200422000883&md=20200425003149_BL

[CR58] Oghuma AP, Libaque-Saenz CF, Wong SF, Chang Y (2016). An expectation–confirmation model of continuance intention to use mobile instant messaging. Telematics and Informatics.

[CR59] Oldfield BM, Baron S (2000). Student perceptions of service quality in a UK university business and management faculty. Quality Assurance in Education.

[CR60] Oliver RL (1981). Measurement and evaluation of satisfaction processes in retail settings. Journal of Retailing.

[CR61] Oliver RL (1977). Effect of expectation and disconfirmation on postexposure product evaluations: An alternative interpretation. Journal of Applied Psychology.

[CR62] Oliver RL (1980). A cognitive model of the antecedents and consequences of satisfaction decisions. Journal of Marketing Research.

[CR63] Oliver RL (1993). Cognitive, affective, and attribute bases of the satisfaction response. Journal of Consumer Research.

[CR64] Palmer SR, Holt DM (2009). Examining student satisfaction with wholly online learning. Journal of Computer Assisted Learning.

[CR65] Park JS, Kim JH, Shin YS (2002). The effects of university education service quality factors on the students’ satisfaction, intention of continuing studies, and word-of-mouth. Asia Marketing Journal.

[CR66] Patricia A (2020). College students’ use and acceptance of emergency online learning due to COVID-19. International Journal of Educational Research Open.

[CR67] Reichheld FF, Schefter P (2000). E-loyalty: Your secret weapon on the web. Harvard Business Review.

[CR68] Rigopoulos G, Askounis D (2007). A TAM framework to evaluate user’s perception toward online electronic payments. Journal of Internet Banking and Commerce.

[CR70] Rogers EM (2003). Diffusion of innovations.

[CR71] Rose, J., & Fogarty, G. J. (2006). Determinants of perceived usefulness and perceived ease of use in the technology acceptance model: senior consumers' adoption of self-service banking technologies. In *Proceedings of the 2nd biennial conference of the academy of world business, marketing and management development: Business across borders in the 21st century* (Vol. 2, pp. 122–129). Academy of World Business, Marketing and Management Development.

[CR72] Sahin I, Shelley M (2008). Considering students’ perceptions: The distance education student satisfaction model. Journal of Educational Technology & Society.

[CR73] Schepers J, Wetzels M (2007). A meta-analysis of the technology acceptance model: Investigating subjective norm and moderation effects. Information & Management.

[CR74] Seddon PB (1997). A respecification and extension of the DeLone and McLean model of IS success. Information Systems Research.

[CR75] Shih HP (2004). Extended technology acceptance model of Internet utilization behavior. Information & Management.

[CR76] Soper, D.S. (2021). A-priori sample size calculator for structural equation models [Software]. Available from https://www.danielsoper.com/statcalc

[CR77] Spreng RA, Mackoy RD (1996). An empirical examination of a model of perceived service quality and satisfaction. Journal of Retailing.

[CR78] Sun PC, Tsai RJ, Finger G, Chen YY, Yeh D (2008). What drives a successful e-learning? An empirical investigation of the critical factors influencing learner satisfaction. Computers & Education.

[CR79] Toquero CM (2020). Challenges and opportunities for higher education amid the COVID-19 pandemic: The Philippine context. Pedagogical Research.

[CR80] Van de Ven AH, Ferry DL (1980). Measuring and assessing organizations.

[CR81] Venkatesh V, Morris MG, Davis GB, Davis FD (2003). User acceptance of information technology: Toward a unified view. MIS Quarterly.

[CR82] Venkatesh V (2006). Where to go from here? Thoughts on future directions for research on individual-level technology adoption with a focus on decision making. Decision Sciences.

[CR83] Venkatesh V, Davis FD (2000). A theoretical extension of the technology acceptance model: Four longitudinal field studies. Management Science.

[CR84] Wang YS, Wang YM, Lin HH, Tang TI (2003). Determinants of user acceptance of internet banking: An empirical study. International Journal of Service Industry Management.

[CR85] Wei HC, Chou C (2020). Online learning performance and satisfaction: Do perceptions and readiness matter?. Distance Education.

[CR86] WHO. (2020). Coronavirus disease (COVID-19) pandemic. World Health Organization. https://www.who.int/emergencies/diseases/novel-coronavirus-2019

[CR87] World Economic Forum. (2020). https://www.weforum.org/agenda/2020/03/coronavirus-china-the-challenges-of-online-learning-for-universities/

[CR88] Zhou L, Wu S, Zhou M, Li F (2020). 'School’s out, but class’ on', the largest online education in the world today: Taking China’s practical exploration during The COVID-19 epidemic prevention and control as an example. SSRN Electronic Journal.

[CR89] Zhu X, Liu J (2020). Education in and after Covid-19: Immediate responses and long-term visions. Postdigital Science and Education.

